# Acute embolic cerebral ischemia as an initial presentation of polycythemia vera: a case report

**DOI:** 10.1186/1752-1947-7-131

**Published:** 2013-05-19

**Authors:** Richard M Zoraster, Richard A Rison

**Affiliations:** 1 Intercommunity Emergency Medical Group, 12401 Washington Blvd., Whittier, CA 90602, USA; 2 Clinical Assistant Professor of Neurology, University of Southern California Keck School of Medicine, Medical Director PIH Health Stroke Program, 12401 Washington Boulevard, Whittier, CA, 90602, USA

## Abstract

**Introduction:**

Patients with polycythemia vera are at high risk for vaso-occlusive events including cerebral ischemia. Although unusual, acute ischemic stroke may be an initial presentation of polycythemia vera. It had been previously assumed that cerebral ischemic events were due to increased blood viscosity and platelet activation within the central nervous system arterial vessels. However, there are now a few isolated case reports of probable micro-embolic events originating from outside of the brain. This suggests unique management issues for these patients.

**Case presentation:**

We present the case of a 57-year-old right-handed Caucasian male in excellent health who presented to the Emergency Department with acute right-handed clumsiness. Hematologic investigations revealed a hyperviscous state and magnetic resonance imaging was consistent with cerebral emboli. Symptoms rapidly improved with phlebotomy and hydration.

**Conclusion:**

The etiology of stroke in polycythemic patients is likely to be multifactorial. While hemodilution has been generally discredited for general stroke management, it is potentially beneficial for patients with polycythemia vera and euvolemic hemodilution should be considered for the polycythemic patient with acute cerebral ischemia.

## Introduction

Acute stroke is the number three cause of death within the United States, and a leading cause of long-term disability [1,2]. There are many populations with an increased incidence of stroke, however patients with polycythemia vera (PV) are a unique subset, both for the pathophysiology and for management. Ischemic stroke may be the first presenting symptom of PV in 15% or more of those affected [3,4]. One study followed 265 patients with PV for a mean of 3 years, with a 5% incidence of stroke [5]. Even among those patient undergoing treatment, the incidence of ischemic stroke is approximately 14.3 per 1000 patient years versus 5.3 per 1000 patient years in the general, over 55 population [5].

Historically, assumptions had been previously made that cerebral ischemia in polycythemic patients was due to the increased viscosity of the blood leading to poor cerebral blood flow, along with platelet (PLT) activation creating an environment for thrombus formation in local cerebral arteries and arterioles [5-7]. While this may be the cause in some patients, recent case reports have suggested another possibility involving formation of emboli.

National efforts have been made to optimize care for all stroke patients, with formation specialized stroke centers [8] adhering to published guidelines for the management of acute ischemic stroke include blood pressure control, anti-PLT agents, and, in appropriate cases thrombolysis [9]. Hemodilution has been considered in multiple past studies, with conflicting results. A meta-analysis has concluded there is a lack of proven efficacy [10], although this was for patients with acute ischemic stroke and did not specifically include patients with hematologic conditions but did state that ‘[h]aemodilution for acute ischaemic stroke should not be used outside of clinical trials with the possible exception of patients with severe polycythemia’.

## Case presentation

A 57-year-old right-handed Caucasian male presented to the Emergency Department with a two-week history of intermittent clumsiness of his right hand. The onset was acute and did not progress. He denied any left-hand difficulties or any problems in his legs. There was no sensory dysfunction, focal weakness, or acute headache. He denied any double vision, blurry vision, difficulty swallowing, or slurred speech and there was no preceding history of any lapses of consciousness or seizure disorder. He had a past history of non-focal headache which he was told were migraines, but these were not coincident with his right-hand complaints. Travel history included a trip abroad where he donated blood and was told that his blood count was high. His wife had recurrently noted redness of his hands and face. Social history was remarkable for the patient being very active, and in fact he had run six miles on the day of presentation. He was a non-smoker, non-drinker, and denied any illicit drug use. Family history was unremarkable for any neurologic or hematologic disease.

Physical exam revealed a healthy appearing male who was afebrile, normotensive and in normal sinus rhythm. Neurological examination demonstrated slight dysmetria of the right hand on finger-to-nose testing. Motor strength testing revealed intact strength throughout without any focal atrophy or abnormal involuntary movements. He could not dial a cell phone with his right hand, but could with his non-dominant hand. Cranial nerves 2–12 were all intact, speech was fluent without aphasia or dysarthria, reflexes were 2/4 throughout, and his gait did not reveal any ataxia. Mental status testing was completely intact. His hands and face were remarkable for dermatologic plethora of mild erythema.

Laboratory evaluation revealed a hemoglobin of 21.3g/dL with a hematocrit value of 61.6%. White blood cell (WBC) count was 6.22 × 10^3^/μL with 75% neutrophils. PLT count was 345 × 1000/μL. Standard autoimmune markers including anti-nuclear antibodies were negative. Factor V Leiden and prothrombin G20210A mutation analysis were both negative. Non-contrast computed tomography (CT) scan of the head showed two focal areas of hypo-attenuation in the left parietal lobe of approximately 11mm in size. A trans-thoracic echocardiogram showed a 55% ejection fraction and no vegetations. A liter of normal saline was bolused over an hour, while arrangements were made to remove two units (500cc’s) of blood. To maintain intravascular volume, a second liter of normal saline was administered. Clopidogril was given orally. Hematologic consultation was obtained and provided the diagnosis of PV. Further blood testing revealed that the patient was positive for the JAK2 V617F mutation.

**Figure 1:**
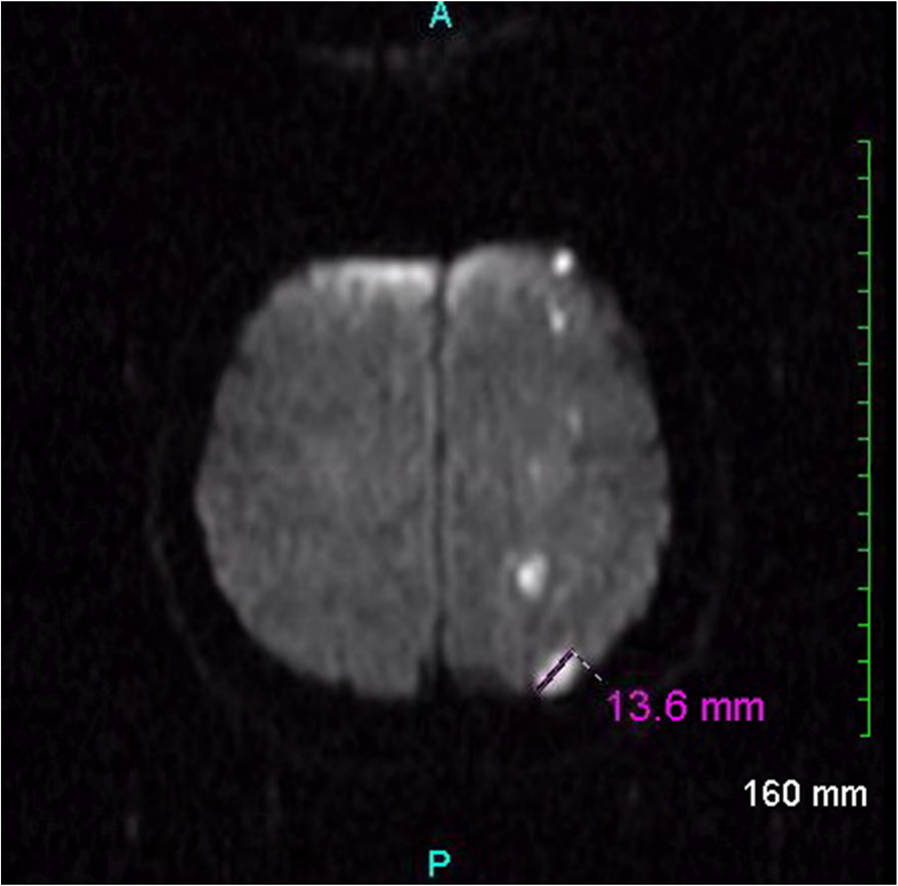
**Magnetic resonance imaging of the brain. **Diffusion-weighted magnetic resonance image of the brain demonstrating numerous small foci of restricted diffusion scattered within the left frontoparietal cortex, subcortical white matter, and centrum semiovale. These foci are consistent with an acute embolic ischemic infarction shower within the left middle cerebral artery distribution.

A brain magnetic resonance imaging (MRI) study with and without contrast revealed 15 to 20 foci of restricted diffusion measuring between 3 and 14mm in size, consistent with an embolic shower in the left middle cerebral artery distribution (Figure 1) confirming the CT scan findings and showing additional areas of involvement. Magnetic resonance angiogram of the brain was normal. Carotid duplex ultrasound found minor plaque in the proximal left internal carotid artery which was not hemodynamically significant. A transesophageal echocardiogram with bubble study performed the day after admission was negative for any patent foramen ovale, atrial septal defect, thrombus, or right-to-left shunt. Cardiac telemetry did not reveal any dysrythmias. An abdominal ultrasound showed mild splenomegaly.

Over the course of his hospital stay, neurologic symptoms gradually improved. Two more units of blood were removed from the patient, and at the time of discharge his hemoglobin level was 15.2g/dL with a hematocrit of 46.9%. He was referred for outpatient hematologic and neurologic follow-ups and to date is doing quite well.

## Discussion

PV is a myeloproliferative disorder resulting in an elevated absolute red blood cell mass because of uncontrolled red blood cell production. This is typically associated with an increase in WBC and PLT production. The increase in WBCs and PLTs is secondary to an abnormal clone of hematopoietic stem cells with increased sensitivity to different maturation growth factors. PV is overall rare, occurring in 0.6-1.6 persons per million population. The peak incidence of PV is 50–70 years of age. However, PV may occur in persons of all age groups, including young adults and children, albeit rarely [11].

The increased hematocrit of PV is the main determinant of blood viscosity. As the viscosity increases, cerebral blood flow decreases [6,7]. PLT marginalization with increased contact to vessel walls occurs, along with local effect of a high hematocrit on vessel walls [6,7]. This fulfils all three components of Virchow’s triad [12], and is consistent with the thought that many strokes in polycythemic patients are due to propagation of a local thrombus [6,7,13,14].

Other mechanisms have been proposed. A 1960 case series from the Mayo Clinic that reviewed PV patients with neurological symptoms discussed the possibility of embolic etiology, but dismissed it because of lack of clinical evidence for a source [13]. This was in the pre-CT, MRI and Doppler era, however, and its conclusions may now be premature in light of present-day neuroimaging. Four prior case reports in adults have documented scattered lesions in an embolic pattern without evidence of patent foramen ovale, cardiac vegetations, or plaque rupture [15-17]. In two of these cases, transesophageal echocardiography demonstrated left atrial “microthrombi” [15,16]. While these were not seen in our case report, the echocardiogram was not obtained until after hemodilution had occurred, which may have resolved the tendency to form thrombi. Furthermore, any cardiac clot may not have been seen since it probably had already embolized prior to our investigations. We therefore postulate that the patient’s PV predisposed him to a prothrombotic state which resulted in an unseen cardiac thrombus which in turn embolized to the cerebrum.

Management of acute ischemic stroke in polycythemia is also unique; it is the only situation where the American Heart Association stroke guidelines suggest a possible value of hemodilution [9]. Additionally, although a meta-analysis of publications did not show a convincing benefit of hemodilution in clinical outcomes of stroke patients in general, the conclusion was that it might be beneficial in polycythemic patients with acute ischemic stroke. If the etiology is the formation of microemboli in the atria due to hyperviscosity, then hemodilution with venisection is likely the most expedient and safest acute treatment.

We feel that there are unique aspects of this case. Firstly, it is example of an acute ischemic stroke due to PV which responded to hemodilution, of which there are few published case reports to date in the literature. Also, it is unusual for PV to present with acute embolic ischemia, as most previously published cases suggest local vessel wall thrombus propagation. We hope that this case reports serves as a reminder of the association of cerebral ischemia and PV.

## Conclusion

Although unusual, acute embolic cerebral ischemia may be an initial presentation of PV. All clinicians involved in the care of stroke patients should be aware of the association of PV and ischemic stroke.

## Consent

Written informed consent was obtained from the patient for publication of this case report and accompanying images. A copy of the written consent is available for review by the Editor-in-Chief of this journal.

## Competing interests

The authors declare that they have no competing interests.

## Authors’ contributions

RMZ and RAR were both involved in clinical diagnostic evaluation and management. RMZ was the initial physician involved in the patient’s care within the Emergency Department and generated the first draft of the manuscript. RAR reviewed and evaluated the neuroimaging studies along with revising and editing the manuscript using an additional literature search. RMZ and RAR were responsible for the intellectual content of the paper. Both authors participated in and provided significant contributions in writing the manuscript. Both authors read and approved the final manuscript.

## Authors’ information

RMZ serves on the editorial board of the *American Journal of Disaster Medicine*. RAR is a Deputy Editor for the *Journal of Medical Case Reports* and is an Associate Neurology Editor for *Case Reports in Neurology*, *Grand Rounds*, and *Webmed Central*.

## References

[B1] Roger VL Go AS Lloyd-Jones DM Adams RJ Berry JD Brown TM Carnethon MR Dai S de Simone G Ford ES Fox CS Fullerton HJ Gillespie C Greenlund KJ Hailpern SM Heit JA Ho PM Howard VJ Kissela BM Kittner SJ Lackland DT Lichtman JH Lisabeth LD Makuc DM Marcus GM Marelli A Matchar DB McDermott MM Meigs JB Moy CS Heart disease and stroke statistics–2011 update: a report from the American Heart Association Circulation 2011 123 4 e18 e209 10.1161/CIR.0b013e3182009701 21160056 PMC4418670

[B2] Carandang R Seshadri S Beiser A Kelly-Hayes M Kase CS Kannel WB Wolf PA Trends in incidence, lifetime risk, severity, and 30-day mortality of stroke over the past 50 years JAMA 2006 296 24 2939 2946 10.1001/jama.296.24.2939 17190894

[B3] Gruppo Italiano Studio Policitemica Polycythemia vera: the natural history of 1213 patients followed for 20 years Ann Intern Med 1995 125 656 664 10.7326/0003-4819-123-9-199511010-00003 7574220

[B4] Hart RG Kanter MC Hematological disorders and ischemic stroke: a selective review Stroke 1990 21 8 1111 1121 10.1161/01.STR.21.8.1111 2202092

[B5] Landolfi R Marchioli R Kutti J Gisslinger H Tognoni G Patrono C Barbui T European Collaboration on Low-Dose Aspirin in Polycythemia Vera Investigators Efficacy and safety of low-dose aspirin in polycythemia vera N Engl J Med 2004 350 2 114 124 10.1056/NEJMoa035572 14711910

[B6] Spivak JL The optimal management of polycythemia vera Br J Haematol 2002 116 2 243 254 10.1046/j.1365-2141.2002.03287.x 11841424

[B7] Spivak JL Polycythemia vera: myths, mechanisms, and management Blood 2002 100 13 4272 4290 10.1182/blood-2001-12-0349 12393615

[B8] Lichtman JH Jones SB Wang Y Watanabe E Leifheit-Limson E Goldstein LB Outcomes after ischemic stroke for hospitals with and without Joint Commission-certified primary stroke centers Neurology 2011 76 23 1976 1982 10.1212/WNL.0b013e31821e54f3 21543736 PMC3109877

[B9] Adams HP Jr del Zoppo G Alberts MJ Bhatt DL Brass L Furlan A Grubb RL Higashida RT Jauch EC Kidwell C Lyden PD Morgenstern LB Qureshi AI Rosenwasser RH Scott PA Wijdicks EF American Heart Association/American Stroke AssociationStroke Council; American Heart Association/American Stroke Association Clinical Cardiology Council; American Heart Association/American Stroke Association Cardiovascular Radiology and Intervention Council; Atherosclerotic Peripheral Vascular Disease Working Group; Quality of Care Outcomes in Research Interdisciplinary Working Group. Guidelines for the early management of adults with ischemic stroke: a guideline from the American Heart Association/American Stroke Association Stroke Council, Clinical Cardiology Council, Cardiovascular Radiology and Intervention Council, and the Atherosclerotic Peripheral Vascular Disease and Quality of Care Outcomes in Research Interdisciplinary Working Groups The American Academy of Neurology affirms the value of this guideline as an educational tool for neurologists Circulation 2007 115 20 e478 e534 10.1161/CIRCULATIONAHA.107.181486 17515473

[B10] Asplund K Haemodilution for acute ischaemic stroke Cochrane Database Syst Rev 2002 4 Art. No.: CD000103 10.1002/14651858.CD000103 12519536

[B11] Besa EC Woermann UJ Polycythemia vera Emedicine 2012 http://emedicine.medscape.com/article/205114-overview. Accessed on 12/25/12

[B12] Bagot CN Arya R Virchow and his triad: a question of attribution Br J Haematol 2008 143 2 180 190 10.1111/j.1365-2141.2008.07323.x 18783400

[B13] Millikan CH Siekert RG Whisnant JP Intermittent carotid and vertebral-basilar insufficiency associated with polycythemia Neurology 1960 10 188 196 10.1212/WNL.10.2.188 14422628

[B14] Landolfi R Di Gennaro L Barbui T De Stefano V Finazzi G Marfisi R Tognoni G Marchioli R European Collaboration on Low-Dose Aspirin in Polycythemia Vera (ECLAP) Leukocytosis as a major thrombotic risk factor in patients with polycythemia vera Blood 2007 109 6 2446 2452 10.1182/blood-2006-08-042515 17105814

[B15] Koennecke HC Bernarding J Diffusion-weighted magnetic resonance imaging in two patients with polycythemia rubra vera and early ischemic stroke Eur J Neurol 2001 8 3 273 277 10.1046/j.1468-1331.2001.00217.x 11328338

[B16] Zimmerman C Walther EU von Scheit W Hamann GF Ischemic stroke in a 29-year-old man with left atrial spontaneous echoes and polycythemia vera J Neurol 1999 246 12 1201 1203 10.1007/s004150050545 10653318

[B17] Segura T Serena J Teruel J Dávalos A Cerebral embolism in a patient with polycythemia rubra vera Eur J Neurol 2000 7 1 87 90 10.1046/j.1468-1331.2000.00008.x 10809920

